# Increased Exploration Capacity Promotes Group Fission in Gregarious Foraging Herbivores

**DOI:** 10.1371/journal.pone.0167516

**Published:** 2016-12-01

**Authors:** Sophie Lardy, Daniel Fortin, Olivier Pays

**Affiliations:** 1 Groupe Ecologie et Conservation des Vertébrés, Université d’Angers, Angers, France; 2 Centre d’Étude de la Forêt (CEF), and Département de Biologie, Université de Laval, Québec, Canada; 3 UMR 6554 CNRS, LETG-Angers, Université d'Angers, Angers, France; University of Tasmania, AUSTRALIA

## Abstract

Many gregarious species display rapid fission-fusion dynamics with individuals frequently leaving their groups to reunite or to form new ones soon after. The adaptive value of such ephemeral associations might reflect a frequent tilt in the balance between the costs and benefits of maintaining group cohesion. The lack of information on the short-term advantages of group fission, however, hampers our understanding of group dynamics. We investigated the effect of group fission on area-restricted search, a search tactic that is commonly used when food distribution is spatially autocorrelated. Specifically, we determine if roe deer (*Capreolus capreolus*) improve key aspects of their extensive search mode immediately after fission. We found that groups indeed moved faster and farther over time immediately after than before fission. This gain was highest for the smallest group that resulted from fission, which was more likely to include the fission’s initiator. Sex of group members further mediated the immediate gain in search capacity, as post-fission groups moved away at farthest rate when they were only comprised of males. Our study suggests that social conflicts during the extensive search mode can promote group fission and, as such, can be a key determinant of group fission-fusion dynamics that are commonly observed in gregarious herbivores.

## Introduction

Animals have developed a broad range of tactics to acquire food [1–3]. Many species, for example, use an area-restricted search when the spatial location of their food is autocorrelated over space [4–5]. This tactic involves a switch between two search modes: an intensive mode that is characterised by low speed and high tortuosity, which enables individuals to remain in food-rich areas, and an extensive mode that allows them to leave poor-quality areas rapidly by increasing their speed and decreasing path sinuosity [6]. The effectiveness of the area-restricted search tactic depends upon the ability of foragers to match their search modes to the spatio-temporal variations in resource availability [7–8]. Accordingly, behavioural decisions that improve the ability of foragers to detect food patches or impeding their movement should directly affect the benefits of using area-restricted search.

Many species from a wide range of taxa are gregarious [9–10], which is a life style that shapes their foraging experience. By joining a large group, foragers should benefit not only from a decrease in predation risk (e.g., dilution effect [11], collective detection [12]), but also from an increase in social information about spatio-temporal patterns in resource availability [13–15]. These benefits, however, can be shadowed by faster resource depletion, higher exploitative and interference competition [16–19], and slower collective movements that are typical of larger groups [20]. Overall, foragers in larger groups should experience a faster decrease in food intake rate (i.e., faster patch depression, *sensu* [21]) and, therefore, have to rely more often upon an extensive search mode, which might be less effective because of the slower movement of large groups. Furthermore, to maintain group cohesion, individuals have to wait until a consensus is reached concerning the movement direction [22–23]. This implies that an individual may experience significant patch depression but still have to wait for other members to be ready to leave the patch. Alternatively, this individual may decide to leave immediately and adopt an extensive search mode. Unless all other group members follow, the individual will have initiated group fission and, at least temporarily, lead the movements of its followers.

At any point in time, each group member may question its membership by assessing its current state of balance between the costs and benefits of remaining part of its group, relative to its expectations following group fission. The individual may have incomplete control over the consequences that group fission has on its intensive search mode. These consequences should depend upon the number of followers, as well as on their identity. In sexually dimorphic ungulates, for example, individuals in unisex groups may be able to carry out activities with greater synchronicity due to shared characteristics (e.g., step length and rate, instantaneous food intake rate, resting bout duration; see [24–25]) compared to individuals of mixed groups [26]). Indeed, males and females often differ in their ecological and physiological needs [27–28], and allometric scaling predicts that female ungulates should move more slowly than males [24, 29]. Such differences could lead to more frequent and stronger conflicts during collective-decision making for members of mixed compared to unisex groups. In turn, the differences should determine the movement characteristics that all group members would adopt to maintain group cohesion (See [30] for the effect of phenotypic assortment in animal socials networks). The onset, speed and sinuosity of the extensive search mode could therefore strongly depend upon group composition.

For any type of social group, the balance between costs and benefits of remaining in the current group should vary dynamically over time and space, most notably following local changes in resource availability. This spatio-temporal dynamic, together with a strong capacity to merge with other groups, can result in high rates of group fission and fusion [31]. Accordingly, many gregarious species are characterised by short-lived associations of conspecifics [32]. Despite decades of investigation, the reason why groups split only to reunite moments later remains an intriguing question [33–37]. The proposition that foragers may leave their current group in an attempt to gain some control over their search tactic (see above) has emerged from inductive inference, based on general behavioural observations [16, 18–20]. The proposition largely rests upon the key assumption that an individual triggers group fission by adopting an extensive search mode, and not simply by pursuing an intensive search mode that ultimately increases the distance between some group members sufficiently to result in group-fission. In other words, the hypothesis would only be supported if, immediately following the fission event, post-fission groups increase their speed and reduce their sinuosity, with the result that their rate of net displacement should be higher than prior to fission. This prediction has never been tested, and, in fact, the influence of social factors (e.g., group size and composition) on the area-search tactic of individuals that are living in fission-fusion societies remains essentially unexplored.

Here, we investigated the effect of group fission upon the characteristics of area-restricted search of free-ranging roe deer (*Capreolus capreolus*), with a particular focus on their extensive search mode. Although the roe deer is a rather solitary forest-dwelling herbivore, individuals become gregarious foragers in open habitats during winter, forming non-permanent groups with short lifetimes, such that an individual generally experiences multiple fission-fusion events within a single day [38]. In winter, mixed-sex groups are generally over-represented, as adult males are no longer territorial [39]. We assessed the immediate changes in exploration capacity (i.e., extensive search mode characteristics) through variations in mean group speed (MGS) and an index of net displacement rate (NDR) over the two moves that preceded and followed fission events. We restricted our analysis to fission events triggered by free departures of individuals (which represented 95% of fission events, see [38]) and not by agonistic interactions. If a gain in exploration capacity motivates animals to leave their initial group, MGS and NDR should be higher after fission rather than before. We also tested for an effect of group composition on the extensive search mode. Based upon sampling of allometric scaling [24]), we would expect groups that are comprised only of males to move faster than other groups. However, because male roe deer are only slightly larger than females (< 10%, [40]), we expect only a small difference in exploration capacity. Functional predictions of the effect of group composition on search mode are uncertain, however, as inter-individual differences on ecological (e.g., energetic needs or diet) and social traits (e.g., inter-individual attraction or avoidance) might also play a role.

## Methods

### Ethics Statement

This study is restricted to behavioural observations of roe deer and, therefore, excludes any animal handling or invasive experiments. Field studies did not involve endangered or protected species. Landowners and the “Communauté de Communes de l’Argonne Ardennaise” gave permission to conduct the study and approved the methodology. The study thus adheres to the “Guidelines for the Use of Animals in Research”, and to the legal requirements of the country in which the work has been carried out.

### Data collection

Data were collected in a natural population of European roe deer near Machault, in north-eastern France (45°25’N, 4°30’E), from December 2002 to February 2003 and from December 2003 to March 2004. Groups of roe deer were observed in an open landscape, which was characterised by an aggregation of large cultivated fields without hedges, from a vantage point that was more than 200 m from the animals. During data collection, cultivated fields offered short crops (mostly wheat (*Triticum spp*.), alfalfa (*Medicago sativa*) and sugar beet (*Beta vulgaris*)) in abundance that were extensively foraged by roe deer. As the study population of roe deer lived under very low natural predation risk, foraging in large groups should allow individuals to increase their opportunities of finding good food patches from social information. During focal sampling, study deer were not disturbed by hunting parties, because the animals were usually hunted in the forest during the winter. Data were recorded from both visual observations and video recordings that were conducted from a four-wheel-drive vehicle. Such methods had already been described and successfully used (see [36]); hence, here we only summarize the approach. Each observation session began early in the morning and a focal group was randomly chosen in the landscape. We focused on scenarios during which a group split into two post-fission groups. Although only few fission events (i.e. 5%) were caused by agonistic interactions between group members [38], we still discarded these observations to specifically study fission events triggering only by free departures of individuals (i.e. 95% of cases). Starting in early morning, a focal group was continuously video-recorded while insuring that all of its members were in the camera's field of view. In the case of group fission, one of the post-fission groups was randomly chosen and continuously video-recorded. Concurrent to video recording, the other post-fission group was visually monitored whenever it got out the camera’s field. From both the video recording and visual observations, we did not observe behaviours indicating the presence of dominance or hierarchy between group members (e.g., inter-individual interference, social conflict). Multiple approaches can be used to identify fission events. Some authors have provided methods to empirically identify distance thresholds beyond which individuals can be considered as belonging to different groups [41]. Ungulate studies [42–43], including in roe deer studies [20, 38, 44], typically consider 50 m as inter-individual distance beyond which group unity has broken up. For consistency with our previous studies, we considered that a fission event has occurred when the distance between at least one individual and the other group members exceeded 50 m. Group size and composition of each initial and post-fission group were noted. Males and females were distinguished on the basis of the presence or absence of antlers, while age class (young *vs*. adult) was determined from individual body size. Young individuals were unambiguously smaller than adults during winter (period of focal sampling), as the former were between 7 and 9 months old.

To investigate group movements, locations of focal groups (approximately from the centre of mass video-recorded or visually monitored) were plotted every five minutes on 1:12 500 aerial photographs; the locations of fission events were also noted. The high-quality photographs and the geometric shapes of the fields allowed the locations of the groups to be determined accurately. Centre of mass in large groups was the location of the individual at the most central position within the group and in small groups an average visual estimation of the gravity centre [38]. As it was impossible to mark (all) individuals in the population, we studied a few numbers of fission events per day on different sites within the study area to limit the risk of individual resampling. Observations (i.e., fission events including one initial group splitting into two post-fission groups) were thus considered to be independent of one another. In this context, 127 fission events were sampled during two three-month sessions, which corresponded to two successive winters.

**Fig 1:**
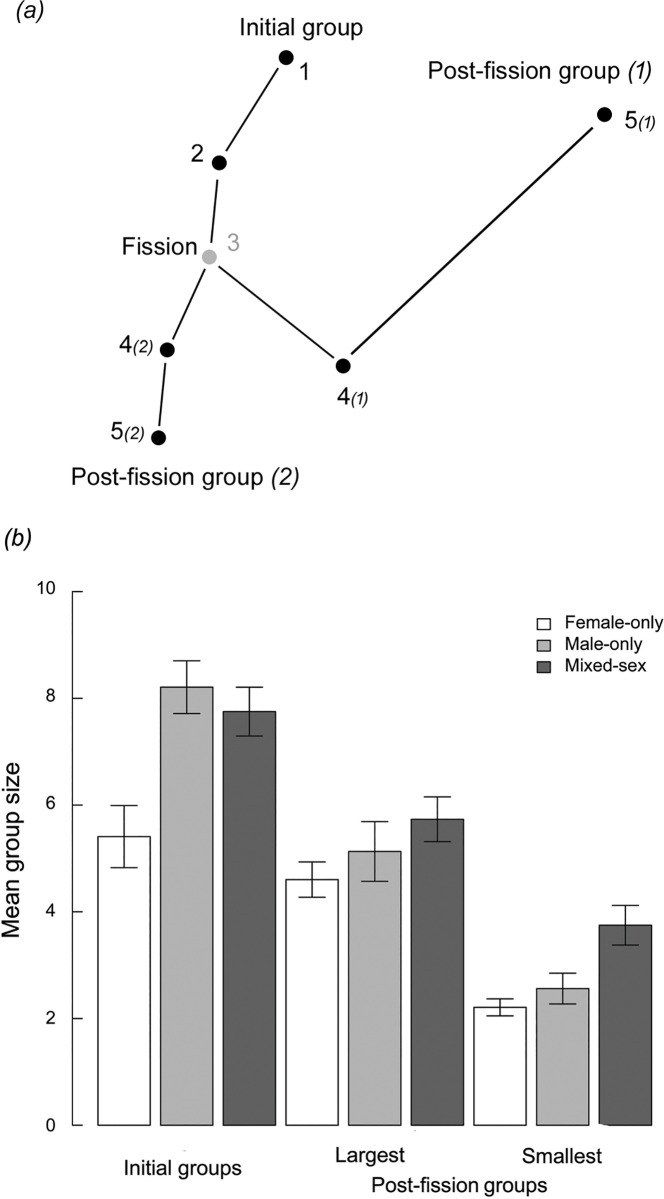
Features of fission events. (a) Example of fission event showing the time-scale (from 1 to 5) at which variation of mean group size and net squared displacement were studied and (b) mean group size (± SE) of initial and post-fission groups according to group composition.

To investigate fine-scale effects of group fission, we compared exploration capacity that was indicated by mean group speed (MGS) and an index of net displacement rate (NDR), which was assessed over the two time moves that preceded and that followed each fission event. Therefore, we considered a restricted window of three locations (i.e., to generate two time moves) before and after fission. This time scale was deliberately chosen to consider the time at which individual decisions operated, i.e., whether gregarious foragers immediately increased the efficiency of their extensive search mode after fission. The location of a fission event was considered as being the last location for the initial group and the first of the two post-fission groups (Fig 1A). All groups were active, i.e., all individuals were foraging, and we did not consider scenarios in which individuals were resting or fleeing. All fission events were unambiguous; they were “passively” generated, i.e., not triggered by inter-individual interference. Indeed, fission events were triggered by foraging individuals that had paths that obviously deviated from or were asynchronised with those of other members in the initial groups. In 68% of fission events, we were able to identify a leader that provoked group fission, and we determined the group (smallest *vs*. largest) in which the leader ended up. Procedures to assess both MGS and NDR over two time moves were calculated from the distance (d) and time (t) between two locations. Using the example of the initial group (*i*) in Fig 1A, MGS_i_ = (d_1-2_+d_2-3_)/(t_1-3_) and NDR_i_ = ((d_1-2_+d_1-3_)/(t_1-3_))^2^. The same formula was used to assess MGS and NDR of post-fission groups.

### Statistical analyses

To investigate patterns in number of individuals in post-fission groups, we compared observed group sizes with sizes expected by random chance alone. We used a Poisson distribution to generate 127 groups with an average of 7 (i.e., the observed mean group size), randomly split into two groups, with individuals having an equal chance of being assigned to either subgroup. This procedure was done 1000 times, and the mean was calculated for the large and small post-fission groups. We then calculated the 95% confidence interval (CI) based on the two distributions comprised of 1000 simulated small and large groups. Significant difference between observed and expected values of post-fission group size was determined based on the 95% CI.

There is a lack of statistical techniques that can readily account for the complex non-independence structure characterizing longitudinal data in highly dynamic systems, such as in fusion-fission societies where group composition changes constantly. If groups were stable, individuals or individual groups could be considered simply as a random factor in mixed-effects models. The problem here is that the association among population members changes constantly–roe deer experiencing several fission-fusion events in a single day [38]–such that non-independence structure rapidly becomes intractable. We thus designed our data collection to minimize the risk of observing multiple times groups comprised of the same members. First, we limited as much as possible the number of observations per day in the same area to avoid resampling the same group more than once in a day. Second, we paid particular attention to study groups in different areas of our study site between two successive days. We thus believe that reasonable inference can be drawn by considering the 127 observed fissions as independent events in our analysis.

We compared MGS and NDR between initial and post-fission groups using linear mixed-effects models including group origin (initial *vs*. post-fission groups), group composition, and the two-way interaction. As group composition can be characterised in three ways, including sex segregation of adults (mixed-sex; male-only *vs*. female-only group), the proportion of adult males (number of adult males/number of adults plus juveniles) or adult females, we first determined which variable best characterised group composition by identifying the candidate models (MGS and NDR) with the lowest AICc (Table 1). We then tested for the effects of each independent variable origin, group composition, and the two-way interaction using *F*-tests for the top-ranking model. Group size was not included as an independent variable in the models because the fission event necessarily implied that initial groups were larger than the two post-fission groups. To account for the dependency between the initial group and the two associated post-fission groups, we included fission event ID as a random factor. We used the same statistical procedure to determine whether or not the effect of the fission on MGS and NDR similarly affected the smallest and the largest post-fission groups.

**Table 1:** AICc-based model selection to investigate the effect of group composition on ln-transformed mean group speed (MGS) and net displacement rate (NDR) using linear mixed-effects models. Models included fission ID as a random effect between initial *vs*. post-fission groups (models including Origin) and between the smallest vs. largest post-fission groups (models including Post-fission group size). Group composition is indicated by either sex segregation (male-only, female-only vs. mixed sex groups) or the proportion of adult males or females.

Model	LogLik.	AICc	*k*
(0a) Null model	-346.393	699.884	3
(1a) Ln(MGS) = Origin + Sex segregation + Origin × Sex segregation	-340.219	697.036	8
(2a) Ln(MGS) = Origin + Proportion of males + Origin × Proportion of males	-343.641	699.682	6
(3a) Ln(MGS) = Origin + Proportion of females + Origin × Proportion of females	-342.870	698.080	6
(0b) Null model	-482.880	971.857	3
(3b) Ln(NDR) = Origin + Sex segregation + Origin × Sex segregation	-471.492	959.581	8
(4b) Ln(NDR) = Origin + Proportion of males + Origin × Proportion of males	-473.726	959.799	6
(5b) Ln(NDR) = Origin + Proportion of females + Origin × Proportion of females	-473.461	959.267	6
(0c) Null model	-243.349	492.842	3
(6c) Ln(MGS) = Post-fission group size + Sex segregation + Post-fission group size × Sex segregation	-236.807	490.503	8
(7c) Ln(MGS) = Post-fission group size + Proportion of males + Post-fission group size × Proportion of males	-239.483	491.478	6
(8c) Ln(MGS) = Post-fission group size + Proportion of females + Post-fission group size × Proportion of females	-238.987	490.485	6
(0d) Null model	-333.566	673.276	3
(9d) Ln(NDR) = Post-fission group size + Sex segregation + Post-fission group × Sex segregation	-323.671	664.231	8
(10d) Ln(NDR) = Post-fission group size + Proportion of males + Post-fission group size × Proportion of males	-328.156	668.823	6
(11d) Ln(NDR) = Post-fission group size + Proportion of females + Post-fission group size × Proportion of females	-327.594	667.701	6

Both MGS and NDR were ln-transformed to linearise the relationships. We performed Tukey’s tests for pairwise multiple comparisons when a two-way interaction was significant. Models were performed with the *lme* function of the *nlme* package and multiple comparisons for parametric models (Tukey contrasts) with the *glht* function of the *multcomp* package in R [45].

## Results

Mean (± SE) group size of initial groups were 7.48 ± 0.33 individuals. We observed an overall asymmetry in group-size of post-fission groups, as the mean (± SE) group size of the largest and smallest groups were 5.20 ± 0.25 and 2.29 ± 0.14 individuals, respectively (See Fig 1B and S1 Table for details of size and composition of initial, pre- and post-fission groups). Both the 95% confidence intervals of the large (5.20–6.03 individuals) and the small (1.48–2.31 individuals) included the values observed in the field. We studied 127 fission events; 86 of these (68%) evidently were triggered by an initiator (i.e., the departure of one individual, which was either followed or not by other group members). Of the 73 cases for which sex was unambiguous, adult females led 49 fissions, while adult males led 13, and young, 11. In three cases, two leaders were observed at the same time and both led to a post-fission group. Altogether, the leaders ended up in 26 of the largest post-fission and 63 in the smallest post-fission groups. Leaders leaving their current groups subsequently tended to form smaller groups.

**Fig 2:**
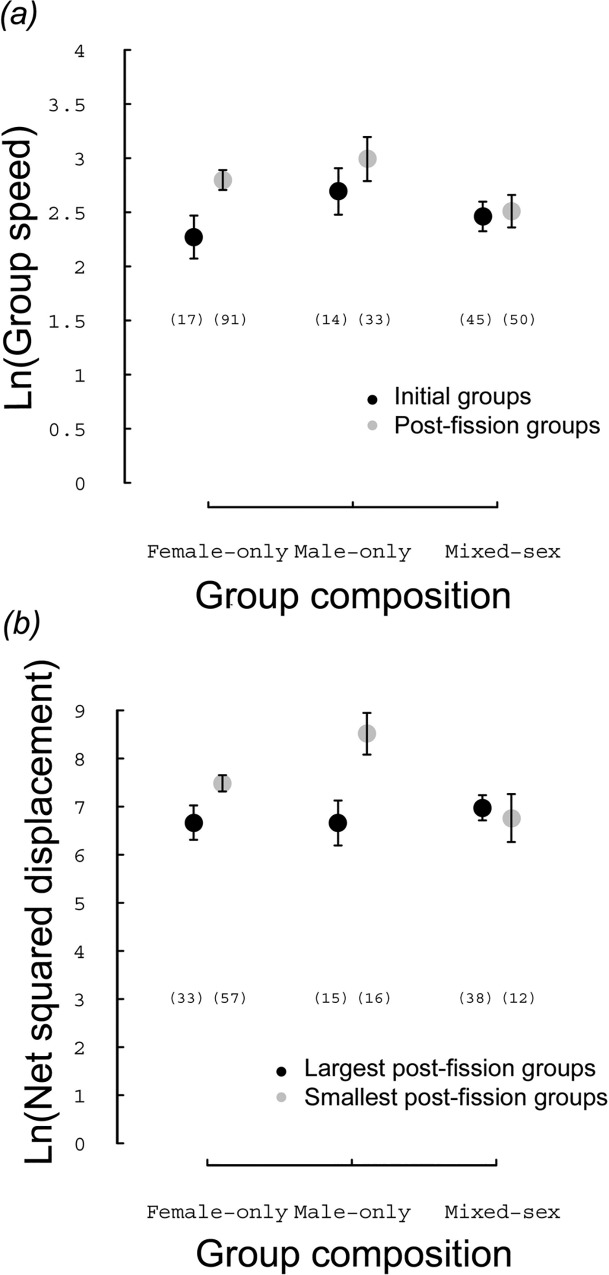
Effect of group composition on exploration capacity. Effect on the ln-transformed (a) mean group speed (m.min^-1^) in initial (black) and post-fission (grey) groups and (b) net squared displacement (m^2^) in the largest (black) and smallest (grey) post-fission groups.

Regardless of group composition (Table 1), we detected a significant difference in both MGS and NDR between initial and post-fission groups, and between the two post-fission groups (Table 2). Post-fission groups moved at a faster rate of speed (Model 1a, Table 2) and farther away over the same time (Model 5b, Table 2) than did initial groups, a trend that was driven mainly by male-only and female-only groups (Fig 2A). Relative to the largest post-fission groups, the smallest groups moved at a faster speed (Model 8c, Table 2) and farther away (Model 9d, Table 2). Group composition did not affect the difference of NDR between the initial and post-fission groups (Model 5b, Table 2). Male-only post-fission groups moved at a faster speed than mixed-sex groups, but at similar speed to female-only groups (Model 1a, Table 2). Finally, group composition did not affect the difference in MGS between the two post-fission groups (Model 8c, Table 2). There was a significant effect of the interaction between group size and group composition on the NDR (Model 9d, Table 2). Pairwise comparisons showed that after fission events (Table 3), smaller post-fission groups of males had a higher NDR than other groups (Fig 2B).

**Table 2:** Model statistics related to the effect of origin (initial *vs*. *post-fission* groups) and post-fission group size (smallest vs. *largest post-fission* groups) on the log-transformed mean group speed (MGS) and net displacement rate (NDR) using linear mixed-effects models including fission ID as a random effect (italicized classes are the reference groups). Models controlled for group composition indicated by either sex segregation (mixed sex, female-only vs. *male-only*), the proportion of adult males or females. Estimates with standard errors are presented when significant.

Model ID	Dependent variable	Independent variable	numDF	denDF	*F*	*P*	β ± SE (P when necessary)
(1a)	MGS	(Intercept)	1	124	1673.475	<0.001	2.999 ± 0.169
		Origin	1	124	3.333	0.070	Initial group: -0.343 ± 0.295
		Sex segregation	2	124	3.510	0.033	Female-only: -0.199 ± 0.195 (0.308), mixed-sex: -0.505 ± 0.217 (0.022)
		Origin × Sex segregation	2	124	1.040	0.357	
(5b)	NDR	(Intercept)	1	126	3953.569	<0.001	7.234 ± 0.180
		Origin	1	126	18.863	<0.001	Initial group: -1.168 ± 0.399
		Proportion of females	1	126	0.018	0.893	
		Origin × Proportion of females	1	126	0.469	0.495	
(8c)	MGS	(Intercept)	1	108	1210.110	<0.001	2.643 ± 0.175
		Post-fission group size	1	59	8.503	0.005	Smallest post-fission group: 0.426 ± 0.223
		Proportion of females	1	59	0.766	0.385	
		Post-fission group size × Proportion of females	1	59	0.001	0.975	
(9d)	NDR	(Intercept)	1	108	2863.441	<0.001	6.644 ± 0.284
		Post-fission group size	1	57	10.332	0.002	Smallest post-fission group: 0.874 ± 0.347
		Sex segregation	2	57	2.021	0.142	
		Post-fission group size× Sex segregation	2	57	3.358	0.042	See Table 3

**Table 3:** Statistics of multiple comparisons (Tukey contrats) on the Ln(NDR) between groups from the linear-mixed effects model 9d (see Table 2). The general linear hypothesis is variable 1 –variable 2 = 0. Significant differences are in bold.

Variable 1	Variable 2	Β	SE	*Z*	*P*
Male-only in largest post-fission group	Female-only in largest post-fission group	0.165	0.499	0.331	0.999
Mixed-sex in largest post-fission group	Female-only in largest post-fission group	0.415	0.384	1.081	0.883
Female-only in smallest post-fission group	Female-only in largest post-fission group	0.975	0.346	2.822	**0.050**
Male-only in smallest post-fission group	Female-only in largest post-fission group	1.914	0.473	4.049	**<0.001**
Mixed-sex in smallest post-fission group	Female-only in largest post-fission group	0.021	0.555	0.038	1.000
Mixed-sex in largest post-fission group	Males-only in largest post-fission group	0.250	0.487	0.512	0.995
Female-only in smallest post-fission group	Male-only in largest post-fission group	0.810	0.459	1.764	0.476
Male-only in smallest post-fission group	Male-only in largest post-fission group	1.749	0.554	3.158	**0.019**
Mixed-sex in smallest post-fission group	Male-only in largest post-fission group	-0.144	0.630	-0.229	1.000
Female-only in smallest post-fission group	Mixed-sex in largest post-fission group	0.560	0.322	1.738	0.493
Male-only in smallest post-fission group	Mixed-sex in largest post-fission group	1.499	0.477	3.143	**0.019**
Mixed-sex in smallest post-fission group	Mixed-sex in largest post-fission group	-0.394	0.540	-0.730	0.977
Male-only in smallest post-fission group	Female-only in smallest post-fission group	0.939	0.453	2.075	0.289
Mixed-sex in smallest post-fission group	Female-only in smallest post-fission group	-0.954	0.527	-1.810	0.445
Mixed-sex in smallest post-fission group	Male-only in smallest post-fission group	-1.893	0.626	-3.025	**0.028**

## Discussion

The search tactic critically links trophic levels by determining the encounter rate between consumers and their resources [46]. Accordingly, there has been strong interest in understanding the link between animal movement and food distribution, including for gregarious species (e.g., [47–48]). Here, we focused on an overlooked aspect of group living: how group fission affects the extensive search mode of area-restricted search in roe deer. We found that deer groups increased both their MGS and NDR immediately following fission, which should increase the efficacy of their extensive search mode [49]. The release in movement constraints that result from group fission could explain the weak group cohesion that has often been reported in many gregarious foragers [50–53]. We know that the probability of observing the fission of a roe deer group increased with group size [38] and that the larger groups are slower [20]. The current study adds to this knowledge by revealing the immediate gain in MGS and NDR that follows a fission event.

The gain in exploration capacity that followed group fission varied among population members, depending upon how they associated with conspecifics. The gain in the extensive search mode that females had experienced after fission depended largely upon the change in group-size. Indeed, when females left their current groups, their gain in NDR was independent of the sex of their followers, as female-only and mixed-sex groups experienced similar increases. Conversely, males increased NDR more strongly when they did not travel with females. We can only speculate about the reasons why males are faster than females. NDR would be higher for males in sexually dimorphic species when they are larger than females [26]. Male roe deer are less than 10% heavier than females [40] and, therefore, sexual dimorphism should not yield strong differences in exploration capacity between males and females [24]. A second mechanism might be based on the social attraction existing between conspecifics in open-membership groups [37] particularly for populations of roe deer that inhabit open agricultural environments [38]. Perhaps males are more likely than females to leave a group in order to travel directly towards (i.e., high NDR) and reach another group. While this hypothesis is still untested, social attraction likely appears to influence group fission of both males and females. The location of foraging conspecifics over the landscape might indicate the presence of rich patches where initiating an intensive search mode might pay off [16]. To increase their chances of finding food-rich patches, individuals may be constantly tempted to join other distant conspecifics, particularly when they can be easily detected, even at long distances [54]. The open landscape characterizing our study area provides deer with a broad perceptual range that allows them to see the location of other groups [38]. Fission-fusion dynamics in roe deer may thus be the result of foragers reacting opportunistically and dynamically to the advantage of leaving the current group to join other foragers that might have discovered food-rich patches. This behaviour may then result in scrounger-producer dynamics (sensu [55]).

Given that the smallest group among those resulting from fission can be familial groups (female-only post-fission groups comprised of one female plus one or two young, see Fig 1B), one could wonder if social factors may have motivated individuals from leaving a group and increased their NDR. However, we did not detect any significant difference of NDR between female-only smallest post-fission groups and mixed-sex smallest or largest post-fission groups; hence, based-family social factors do not seem to be the main reason the observed differences in MGS and NDR between the two post-fission groups.

Deer form open membership groups in open landscapes [38], for which group composition of post-fission groups appears uncertain. We found, however, that group size may be predictable to some degree, at least qualitatively, because most fission initiators ended up in the smallest post-fission groups. Consequently, the leaders should experience the strongest gain in their extensive search mode. In several species, including the domestic goose *Anser domesticus* [56] or the white-faced capuchin *Cebus capucinus* [57], animal aggregation is associated with temporary synchrony of activities between group members and individuals following movement of a (non-permanent) leader or its proximate neighbours. Here, fission describes a passive process under which cohesion (i.e., spatial distribution and orientation of group members) is disrupted. Nevertheless, other studies in more structured or hierarchical groups have suggested that leaders may display distinct behaviours to encourage or force other group members to follow [58]. For instance, in the rhesus macaque *Macaca mulatta*, initiators increase their probability of being followed when they performed numerous back-glances [59]. To what extent deer are able to detect potential followers before fission or even control to some degree their numbers by adjusting their speed and direction remains unclear.

Despite strong evidence that food availability can affect fission-fusion dynamics [41, 60–61], the possibility that individuals may leave a group to increase their foraging efficiency has remained poorly documented. Our study suggests that social conflicts during the extensive mode of area-restricted search can trigger group fission, and as such, be a key determinant of group fission-fusion dynamics that are commonly observed in gregarious herbivores. The task might now be to investigate how predation risk and food distribution influence group fission when populations are exposed to high predation rates. Future work should also determine if foraging strategies involving group fusion events are associated with an immediate increase in intake rate for individuals that join a new group.

## Supporting Information

S1 Table Features of groups involved in fission. FissionID and GroupID are the fission identity and Group identity respectively. Origin indicates whether the group is the initial pre-fission group or one of the two post-fission groups (i.e. PostF1 or PostF2). gp_size is group size and nM, nF, nY is the number of adult males, females and young respectively. sex_segr differentiates female-only (onlyF), male-only (onlyM) and mixed-sex groups (mixed). MGS and NDR are the mean group speed and net square displacement. NA corresponds to missing value.
(PRN)
